# PI-3K Inhibitors Preferentially Target CD15+ Cancer Stem Cell Population in SHH Driven Medulloblastoma

**DOI:** 10.1371/journal.pone.0150836

**Published:** 2016-03-03

**Authors:** Alok R. Singh, Shweta Joshi, Muamera Zulcic, Michael Alcaraz, Joseph R. Garlich, Guillermo A. Morales, Yoon J. Cho, Lei Bao, Michael L. Levy, Robert Newbury, Denise Malicki, Karen Messer, John Crawford, Donald L. Durden

**Affiliations:** 1 Department of Pediatrics, Moores Cancer Center, UC San Diego Health System, La Jolla, CA, United States of America; 2 SignalRx Pharmaceuticals, San Diego, CA, United States of America; 3 Departments of Neurology and Neurosurgery, Stanford University School of Medicine, Stanford, CA, United States of America; 4 Biostatistics Department, Moores Cancer Center, UC San Diego Health System, La Jolla, CA, United States of America; 5 Department of Neurosurgery, UCSD Rady Children’s Hospital, La Jolla, CA, United States of America; 6 Department of Pathology, UCSD Rady Children’s Hospital, La Jolla, CA, United States of America; 7 Department of Neurosciences Division of Child Neurology, UCSD Rady Children’s Hospital, La Jolla, CA, United States of America; 8 Division of Pediatric Hematology-Oncology, UCSD Rady Children’s Hospital, La Jolla, CA, United States of America; University of Navarra, SPAIN

## Abstract

Sonic hedgehog (SHH) medulloblastoma (MB) subtype is driven by a proliferative CD15+ tumor propagating cell (TPC), also considered in the literature as a putative cancer stem cell (CSC). Despite considerable research, much of the biology of this TPC remains unknown. We report evidence that phosphatase and tensin homolog (PTEN) and phosphoinositide 3-kinase (PI-3K) play a crucial role in the propagation, survival and potential response to therapy in this CD15+ CSC/TPC-driven malignant disease. Using the ND2-*SmoA1* transgenic mouse model for MB, mouse genetics and patient-derived xenografts (PDXs), we demonstrate that the CD15+TPCs are **1**) obligately required for SmoA1Tg-driven tumorigenicity **2**) regulated by PTEN and PI-3K signaling **3**) selectively sensitive to the cytotoxic effects of pan PI-3K inhibitors *in vitro* and *in vivo* but resistant to chemotherapy **4)** in the SmoA1Tg mouse model are genomically similar to the SHH human MB subgroup. The results provide the first evidence that PTEN plays a role in MB TPC signaling and biology and that PI-3K inhibitors target and suppress the survival and proliferation of cells within the mouse and human CD15+ cancer stem cell compartment. In contrast, CD15+ TPCs are resistant to cisplatinum, temozolomide and the SHH inhibitor, NVP-LDE-225, agents currently used in treatment of medulloblastoma. These studies validate the therapeutic efficacy of pan PI-3K inhibitors in the treatment of CD15+ TPC dependent medulloblastoma and suggest a sequential combination of PI-3K inhibitors and chemotherapy will have augmented efficacy in the treatment of this disease.

## Introduction

Medulloblastoma (MB) is an aggressive cerebellar tumor and the most common pediatric brain malignancy [1, 2]. The current treatment for medulloblastoma includes resection of the tumor followed by radiation and chemotherapy which includes cisplatinum regimens. Although the cure rate is 50–80%, survivors suffer severe side effects including growth impairment, endocrine disorders, and marked neurocognitive deficits [3]. Thus, more effective and less toxic therapies for medulloblastoma are urgently needed. Recently, several groups [4–8] have performed gene expression profiling and DNA-copy-number analysis of MB, and have identified at least four major subtypes of the disease: WNT, Sonic hedgehog (SHH), Group C, and Group D. These molecular subtypes have distinct characteristics in terms of gene expression, mutational profiles, epidemiology, and prognosis. Among molecular subtypes, tumors associated with uncontrolled activation of SHH pathway are commonly defined as SHH MB. The SHH pathway is an essential embryonic signaling cascade that regulates stem-cell and progenitor-cell differentiation in multiple developmental processes [9]. Mutations in the SHH pathway suppressor *Patched* or alterations of other SHH pathway components result in its permanent activation and MB tumor formation [10, 11]. About 30% of MB exhibits uncontrolled activation of the SHH signaling pathway [11]. Although, several smoothened (SMO) antagonists including NVP-LDE225 & GDC0449 are currently being evaluated in clinical trials in patients with medulloblastoma, there is rapid development of tumor resistance [12, 13]. A study by Buonamici et al demonstrated that NVP-LDE225 resistance in MB is mediated by the activation of the phosphoinositide 3-kinase (PI3K) signaling pathway. [14]. Existing literature suggests that the tumor suppressor, PTEN and its target PI-3K are important in the pathogenesis of SHH-associated MB [15–20]. Recent genomic analysis of medulloblastoma tumors revealed that PI-3K mutation (PIK3CA, PTEN, PIK3C2G) is frequent in SHH subgroup tumors [21, 22]. In one of the studies, out of 13 Hedgehog subgroup tumors profiled, 2 had loss-of-function mutations in *PTEN*, and another patient had an activating mutation in PIK3CA [22]. In another study, out of 133 SHH MB tumors profiled, PI-3K pathway is mutated in >5% of SHH MBs [21, 22].

Multiple reports indicate that MB is driven by treatment resistant stem cell-like cells, termed cancer stem cells/tumor propagating cells (CSC/TPCs). Landmark studies demonstrate that tumor samples extracted from murine genetic MB models, Sonic hedgehog (*SHH-Patched*) and from human MB, are propagated by cells expressing the progenitor marker CD15/SSEA [23, 24]. CD15 is a carbohydrate antigen that is expressed on both progenitors and stem cells in the embryonic and adult central nervous system [25, 26] and most notably is considered as an important marker for TPCs in the SHH subgroup of medulloblastoma [23].

TPCs are considered to be proliferative and a major contributor to tumor resistance and recurrence [27–30]. Several studies have demonstrated a role for the PI-3K/AKT pathway in proliferation and propagation of TPCs [31–33]. Reports have shown that blocking PI-3K activity suppresses the proliferation of TPCs in breast, ovary and various other cancers [34]. Hambardzumyan *et al*. suggested that the PI-3K pathway activity regulates survival of cancer stem cells following radiation in medulloblastoma *in vivo*. [35]. Thus, we hypothesized that targeting the PTEN-PI-3K signaling axis in the MB TPC compartment may provide long-term tumor control and/or eradication of medulloblastoma. Previously, our group has reported that heterozygosity of PTEN promotes tumorigenesis in both human and in the *SmoA1* mouse model of medulloblastoma [15]. We reported that 61% of human medulloblastoma tumors have lost expression of the PTEN protein and this loss in PTEN is of prognostic significance in this disease (15). Herein, using the *SmoA1Tg* mouse model and primary human MB patient xenograft tumor samples (PDXs), we observed that tumor-propagating capacity of CD15+ TPCs in SHH-driven MB is regulated at least in part by the PTEN-PI-3K signaling pathways, and that targeting this axis using PI-3K inhibitors may block the *in vivo* propagation of TPCs and induce apoptosis.

## Materials and Methods

### Animal studies

ND2:*SmoA1* (*SmoA1*) transgenic mice were a gift from James Olson (University of Washington, Seattle, WA) [36]. Mice were maintained in the Moores Cancer Center vivarium at University of California, San Diego, and all experiments were performed using procedures approved by the University of California, San Diego IACUC committee.

### Stereotaxic implantation of tumor cells

Stereotaxic implantation of tumor cells was performed as described before [23, 37]. Nude *nu-nu* mice were anesthetized using 60 mg/kg ketamine (Fort Dodge Animal Health) plus 20 mg/kg xylazine (Ben Venue Laboratories), and positioned in a stereotaxic frame with a mouse adapter (Kopf Instruments). An incision was made in the midline of the scalp over the cerebellum, and a small hole was made in the skull (3 mm to the right and 1 mm anterior to bregma) using a beveled (sharp point) 25 G needle. A 30-gauge Hamilton syringe loaded with cells was mounted on a micromanipulator and introduced through the hole to the surface of the right frontal lobe, at a depth of 4.5 mm. Freshly-sorted CD15+/- tumor (uncultured) cells were injected over the course of 2 minutes, and the needle was left in place for five more minutes to avoid reflux. Finally, the skin was closed with 6–0 fast absorbing plain gut suture using a 3/8 PC-1 cutting needle (Ethicon). Animals were monitored continuously during and in postoperative period to assure that mice have recovered from surgery and are ambulatory without evidence of discomfort. Potential painful and stressful effects of this survival surgery include: 1) poor feeding, 2) weight loss, 3) ruffled fir, 4) loss of mobility in cage, 5) evidence of infection at surgical site. The analgesic buprenorphine (0.1 mg/kg) was injected in case of pain or discomfort. Nonabsorbable sutures and/or staples was removed 10–14 days following surgery. If morbidity is not corrected by our interventions, mice were euthanized using CO2 followed by cervical dislocation.

### Cell isolation & flow cytometry

Tumor cells were isolated from PDX as well as 4 to 6-month-old SmoA1Tg mice as follows. Briefly, tumor tissue was cut into small pieces, and incubated at 37°C for 30 min in digestion buffer consisting of Dulbecco’s PBS (DPBS, Life Technologies, Grand Island, NY) with 10 U/ml papain (Worthington, Lakewood, NJ), 200 μg/ml L-cysteine, and 250 U/ml DNase (Sigma, St. Louis, MO). The digestion buffer was then removed and replaced with DPBS containing 8 mg/ml soybean trypsin inhibitor (Boehringer Mannheim, Indianapolis, IN), 8 mg/ml bovine serum albumin (BSA, Sigma), and 250 U/ml DNase, followed by titration of tissue using pipettes of decreasing bore size to obtain a single-cell suspension. Cells were centrifuged at room temperature and resuspended in PBS containing 200 μg/ml BSA (PBS/BSA) and passed through a cell strainer (Becton Dickinson, Franklin Lakes, NJ) to remove debris. This suspension was centrifuged through a step gradient of 35% and 65% Percol (Amersham Biosciences), and cells were harvested from the 35%-65% interface, washed in PBS/BSA. For sorting of CD15+ and CD15- cells, tumor cells were re-suspended in FACS buffer (DPBS + 2% FBS) and stained for 30 min with CD15 antibody (BD Biosciences, Cat no. 340850, primary antibodiy) washed with FACS buffer, stained for 30 minutes with secondary antibodiy (FITC), and then analyzed or sorted using a FACSVantage SE flow cytometer.

### Cell proliferation, BrdU incorporation, apoptosis and cell cycle analysis

Tumor cells were isolated as previously described [23] from human patient derived xenografts (PDX) as well as 4 to 6-month-old *SmoA1* PTEN+/+ mice displaying physical and behavioural signs of medulloblastoma.

FACS sorted CD15+ and CD15- cells were plated at 4 x 10^4^ cells/well in ultralow binding 96-well plates in serum-free medium containing Neurobasal and B27 supplements. Cells were incubated overnight and treated with DMSO or inhibitors for 48 hr. Cell viability assay was performed using AlamarBlue® (Roche) according to manufacturer’s protocol. For BrdU incorporation studies, tumor cells were isolated from Smo A1 Tg model as described above. 2 million tumor cells per well were plated into 24-well plates in serum-free medium containing Neurobasal and B27 supplements. The cells were pulsed with BrdU for 30 minutes and then washed with media to remove any remaining BrdU. Cells were collected immediately after the pulse (“30 minutes”) and stained with CD15 antibody as described above. The cells were then fixed and stained using the FITC BrdU Flow Kit (BD Biosciences) and propidium iodide (PI) according to the manufacturer’s instructions. The analysis was performed using a FACS Calibur flow cytometer (BD Biosciences). For apoptosis studies, CD15+ and CD15- cells were treated with inhibitor for 24 hrs, followed by caspase-3 activity assay using kit (Roche) or staining with annexin V^FITC^ antibody and propidium iodide (PI) according to manufacturer's instructions (BD, Pharmingen, San Diego, CA). For cell cycle analysis DNA content was analyzed with FACS Calibur flow cytometer (BD Biosciences).

### Western blot analysis

FACS sorted CD15+ or CD15- cells were treated with different concentrations of inhibitors or DMSO for 30 minutes and then stimulated with IGF 50 ng/ml for 30 minutes. Cells were lysed with RIPA lysis buffer (Pierce) containing protease inhibitor cocktail. Proteins were quantitated by the BCA protein assay (Pierce) and equal amounts of protein were resolved by polyacrylamide gels, transferred to nitrocellulose membrane and probed with following primary antibodies: p-AKT(Ser473) (cat no. 9271), p-AKT(Thr308) (cat no. 9275), AKT(cat no. 9272), p-P70S6K(Thr389) (cat no. 9205), p70S6K (cat no. 2708), p-4EBP1(Thr37/46) (cat no. 2855), 4EBP1(cat no. 9452), pERK(Thr202/Tyr204) (cat no. 9101), p-PRAS40(Thr246) (cat no. 2997), PRAS40 (cat no. 2610), p27 Kip1 (cat no. 2552), p-MDM2(S166) (cat no. 3521), Bad (cat no. 9292) and PARP(cat no. 9542) (all from Cell Signaling Technologies); p21 Cip1/Waf1 (sc-397), Bax (sc-493) and β-actin (sc-47778) from Santacruz.

### Quantitative real-time analysis

RNA was extracted from CD15+ and CD15- cells using RNeasy Kit (Qiagen, Germantown, MD) according to manufacturer’s instructions. For RTPCR, cDNA was prepared from 1 μg RNA sample using iscript cDNA synthesis kit (Bio-Rad, Hercules, CA). cDNA was amplified by RT-PCR reactions with 1× SYBR green supermix (Bio-Rad, Hercules, CA) in 96-well plates on an CFX96 Real time system (Bio-Rad, Hercules, CA) using different primers for human or mouse genes. The sequence of the primers are as described in S1 Table. Relative expression levels were normalized to GAPDH expression according to the formula < 2^(Ct gene of interest-Ct GAPDH) >.

### *In-vivo* BKM120 treatment

To study effect of BKM-120 on tumor growth *in vivo*, CD15+ and CD15- TPC were implanted intracranially into the cerebella of secondary NSG mice or subcutaneously in nu-nu mice. For subcutaneous tumors, 20 days after transplantation, mice were randomly separated into two groups: Group 1 was given vehicle (untreated) and Group 2 was given 30 mg/kg BKM-120 by oral gavage once daily for 21 days. Tumor dimensions were recorded every third day and tumor volume was measured using the following formula: volume = 0.5 x length x (width)^2^. For intracranial tumors, 40–50 days after implantation, tumors were checked by MRI and divided into groups and treated as described for subcutaneous tumors. Measurement of tumor volume for subcutaneously implanted tumor was done by callipers and for intracranial implanted tumor was performed by Magnetic resonance imaging (MRI). MRI was performed using a 1.0-T MRI. Mice were anesthetized with 2% isofluorane and the mice were then imaged with an Aspect M2 1.0-Tesla small animal scanner (Aspect Imaging; Shoham, Israel). T2-weighted, images were obtained by using a repetition time of 2500 ms, an echo time of 60 ms, a slice thickness of 1 mm, field of view of 35 mm and a matrix size of 184 x 184 (in plane resolution of 35/184 = 0.19mm). For MR imaging studies, tumor volumes were measured by manually segmenting tumors using either Varian's Image Browser software or the public domain program ImageJ Image (http://rsb.info.nih.gov/ij). T2-weighted images were sometimes used to help clarify tumor margins.

### Human tumor isolation and propagation

Human MB tissue for patient-derived xenografts was obtained from surgical resection of tumors at Rady Children’s Hospital (San Diego, CA). All procedures using human tissue were approved by the Institutional Review Boards of Rady Children’s Hospital. Upon retrieval, the tissue was mechanically dissociated into a single-cell suspension, then immediately injected into the brain of NSG mice. When the mice became symptomatic, the tumors were again dissociated into single-cell suspensions and then re-transplanted back into the brain of naïve hosts to establish a propagated line for each patient-derived xenograft.

### Microarray analysis

*SmoA1Tg* tumor cells were sorted into CD15+ and CD15- populations and used for RNA isolation using RNeasy Kit (Qiagen, Germantown, MD). RNA integrity was assessed using an Agilent 2100 Bioanalyzer. Samples showing RNA integrity (RIN) greater than 8.0 were used for microarray analysis. RNA was labelled and hybridized to Affymetrix Mouse Genome 1.0 ST arrays. The array data were processed by the RMA software. Significance Analysis of Microarray (SAM) software was used to determine differential expression with a false discovery rate (FDR) <1% and a minimum fold-change of 2 unless otherwise stated. Heatmaps were generated using the R package. Gene expression data were transformed into Z-score and hierarchical clustering with an Euclidean distance was applied to generate the row and column dendrograms. Microarray data have been deposited in the GEO public database (http://www.ncbi.nlm.nih.gov/geo/), with GEO accession number is GSE41717.

### Subclass and SUBMAP analysis to compare murine CD15+ TPCs to human MB subgroups

MB subclass and SUBMAP analysis, which allows for cross-platform and cross-species comparison of microarray data based on Kolmogorov-Smirnov statistics (33), was used to compare murine tumors to the human MB samples described in [4]. The Subclass Mapping module in the Gene Pattern software package (www.broadinstitute.org/genepattern) was used to compare the mouse dataset to a gene expression dataset composed of primary human MB classified into molecular subtypes (c1–c6) as defined in Cho et al. and a series of normal cerebellum samples and atypical teratoid rhabdoid tumors [4]. Mapping results are represented as a subclass association (SA) matrix-filled with p-values for each subclass association.

### Gene set enrichment analysis (GSEA)

GSEA was carried out as described in [38]. Briefly, genes were ordered based on their differential expression between two classes (CD15+ vs. CD15-). An enrichment score (ES) was then calculated for each gene set. Gene sets with a nominal p-value < 0.05 were included as significant. For this analysis, curated gene sets (c2) from MSigDB v.3.0 (http://www.broadinstitute.org/gsea/msigdb/index.jsp) were utilized. PCA analysis of the 22 leading edge genes identified in the SUBMAP evaluation were compared across species barrier in CD15+, CD15- and in human MB tumor gene expression database [4].

## Results

### PI-3K signaling is highly elevated in CD15+ TPCs isolated from *SmoA1 Tg* medulloblastoma mouse model

**Fig 1:**
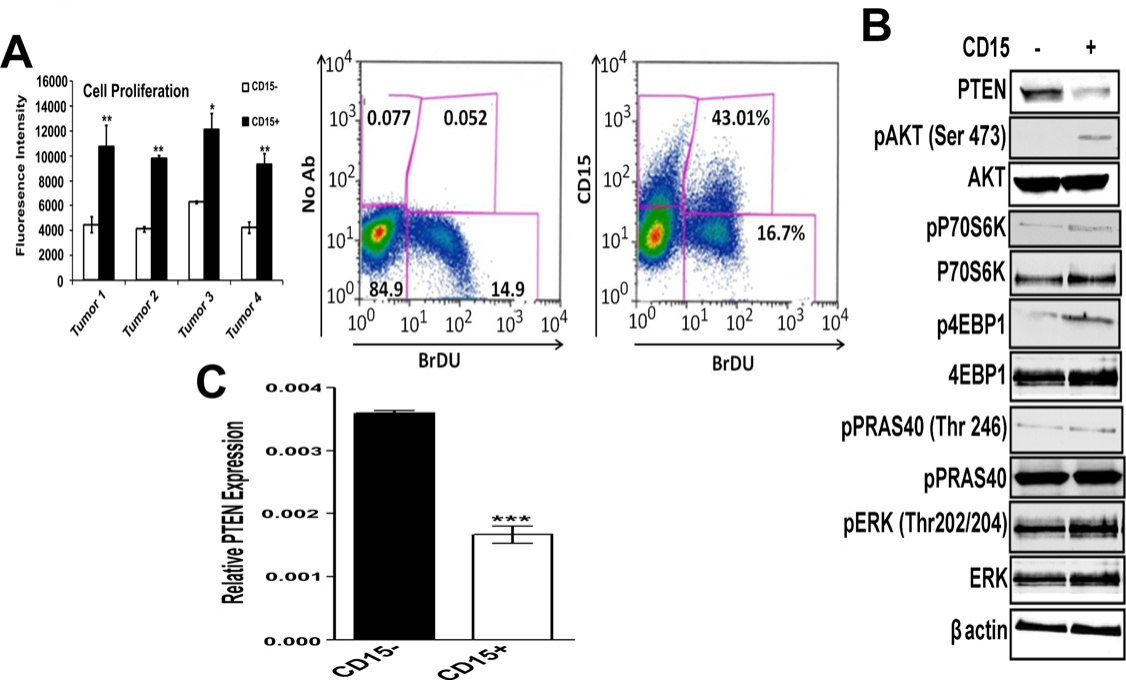
CD15+ TPCs isolated from *SmoA1* Tg medulloblastoma mouse model are highly proliferative and shows elevated PI-3K signaling (A) Left panel shows proliferation of CD15+ and CD15- cells isolated from *Smo A1* tumors. FACS sorted CD15+ and CD15− tumor cells (n = 4) were cultured for 48 hours in serum-free medium, followed by addition of AlamarBlue® and incubation of plates at 37°C in 5% CO_2_ for 6 hours. Fluorescence signals were read as emission at 590 nm after excitation at 560 nm. Right panel shows BrDU incorporation in CD15+ and CD15- cells isolated from the Smo A1 Tg. CD15+ and CD15- from Smo tumors were pulsed with BrDU as described in Materials and Methods. (B) FACS sorted CD15+ and CD15- cells were analyzed for expression of PTEN and phosphorylation of Akt, 4EBP1, P70S6K and Erk. β-actin was used as a loading control. (C) Quantitative PCR analysis of mRNA for expression of PTEN in FACS sorted CD15+ and CD15- cells (n = 3). Relative expression levels were normalized to GAPDH expression. Experiment was repeated 3 times with similar results.

It is well established that CD15 is a marker for tumor propagating cells in Ptc+/- model of SHH driven medulloblastomas [23]. In the present study, *ND2SmoA1* transgenic mouse model was used to characterize the signaling pathways required for proliferation of CD15+ TPCs. First, we performed preliminary experiments in our *SmoA1* model to validate that CD15 is a cell surface marker for tumor propagating cells in this SHH driven medulloblastoma model. For this purpose, an orthotopic transplantation assay was established in which *SmoA1* PTEN+/+ tumor cells were sorted into CD15+ and CD15− fractions, and 2 x 10^6^ cells from each fraction were stereotaxically implanted into the cerebellum of nude/*nu-nu* mice. S1A Fig shows the FACS data validating that pure CD15+ population is isolated from the tumors. As shown in S1B Fig, implantation of only CD15+ TPCs resulted in secondary tumors within 10–12 weeks that histologically resembled the primary tumors from which the cells were derived (data not shown). In S1B Fig, we demonstrate Ki67 staining only in secondary tumors derived from CD15+ TPCs indicating the higher proliferative index of these cells relative to normal brain. Moreover, our findings demonstrate that **only** CD15+ cells from *SmoA1 PTEN+/+* medulloblastoma can generate tumors *in vivo*. In order to further evaluate the stem cell like properties of CD15+ TPC, we performed real time PCR analysis of a number of stem cell markers and the results demonstrate that certain stem cell markers e.g. oct4, klf4, sox2, cxcr4, pou5f1, nanog, nestin and musashi are highly enriched in the CD15+ TPC compartment in the *SmoA1*Tg mouse model (S1C Fig). In order to gain further insight into the tumor propagating properties of CD15+ cells, we performed a number of biochemical and genomic analyses of CD15+ and CD15- cell populations isolated from *SmoA1*Tg tumors. Based on this analysis, we found that the CD15+ population isolated from Smo*A1* Tg mouse model form neurospheres (data not shown), display a distinct expression pattern and are highly proliferative as revealed by increasing viable cell numbers over time (Fig 1A, left panel) and higher BrDU incorporation in CD15+ cells as compared to CD15- cells from the same tumor (Fig 1A, right panel). This result is also supported by higher H^3-^thymidine incorporation (data not shown) in CD15+ cells as compared to CD15- cells. Data presented in S2 Table and S2 Fig shows that genes related to proliferation and cell survival (S2A Fig), SHH signaling pathway (S2B & S2C Fig) and angiogenesis (S2D Fig) are highly elevated in the CD15+ population. Overall, these data indicate that the tumor-propagating capacity of CD15+ cells is associated with an increased capacity to proliferate and a decreased tendency to undergo apoptosis and differentiation.

PI-3K/AKT pathway has been shown to be important for the proliferation of TPCs in both solid tumors and leukemia [31–33]. In order to examine the potential mechanistic role for the PI-3K signaling pathway in TPC propagation and survival in medulloblastoma, we determined the relative activation state of PI-3K/AKT in CD15+ TPCs vs. CD15- non-TPCs. Western blot analysis revealed that CD15+ cells have lower basal levels of expression of PTEN and have an activated PI-3K signaling axis compared to CD15- cells, showing substantial increase in phospho-AKT, phospho-S6 and phospho-4EBP1 (Fig 1B). These results were supported by the augmented levels of PTEN mRNA detected in CD15- population as compared to the CD15+ cells (Fig 1C). Taken together, these results suggest that PTEN expression is downregulated and PI-3K signaling is elevated in CD15+ TPC as compared to CD15- population.

### Preferential targeting of TPC by PI-3K inhibitors *in vitro*

**Fig 2:**
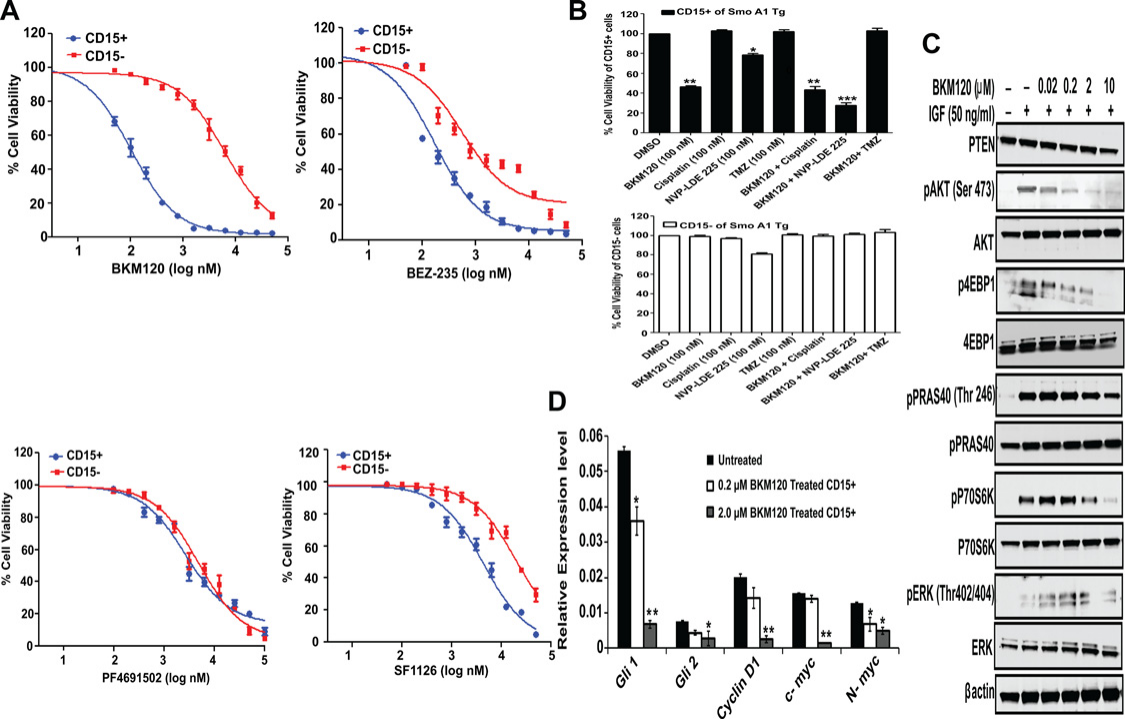
Preferential targeting of TPCs by PI-3K inhibitors *in vitro* (A) Effect of PI-3K inhibitors on proliferation of CD15+ and CD15- cells. CD15+ and CD15- cells were cultured in serum-free media containing no additive, DMSO (vehicle), BKM-120, BEZ-235, PF-04691502, and SF1126 at different conc. After 48 hr, AlamarBlue® was added and plates were incubated at 37°C in 5% CO_2_ for 6 hours. Fluorescence signals were read as emission at 590 nm after excitation at 560 nm. (B) CD15+ and CD15- cells were treated with 100nM conc. of cisplatinum, TMZ, NVP-LDE-225 either alone or in combination with BKM 120 and analyzed for cell viability using Alamar Blue. (C) CD15+ TPCs were treated with BKM120 for 30 minutes followed by stimulation with IGF (50 ng/ml). Cell lysates were analyzed by Western blot for phosphorylation of substrates of PI-3K signalling. (D) Relative expression of SHH pathway genes in BKM120 treated (0.2, 2.0 μM) and untreated CD15+ TPCs. Relative expression levels were normalized to GAPDH. Graphs present mean ± SEM of 3–4 mice in each group for B and D. Statistical significance is assessed by two sample *t*-test where *denotes *P*<0.05, ** denotes *P*<0.01 and *** denotes *P*<0.001. Experiment was repeated 4–5 times with similar results.

The above results demonstrate that PI-3K signaling is upregulated in CD15+ TPC as determined by the lower levels of PTEN and the activation of AKT. Hence, we hypothesized that treating CD15+ cells with PI-3K inhibitors would preferentially block the proliferation of CD15+ TPCs. For this, a panel of PI-3K inhibitors, SF1126 [39], BEZ-235 [40] (Selleck chemicals), PF4691502 [41] (Selleck chemicals) and BKM120 [42] (Novartis) were used. Cisplatinum, TMZ and NVP-LDE-225 were purchased from Selleck Chemicals. Results in Fig 2A show that although PI-3K inhibitors dose dependently reduce the proliferation of both CD15+ and CD15- TPCs isolated from *SmoA1* Tg mouse model, it was more potent against CD15+ population. The IC_50_ for BKM120, BEZ, PF4691502 and SF1126 is 0.156 μM, 0.167 μM, 2.6 μM and 4.3 μM for CD15+ cells and 6.17 μM, 5.54 μM, 4.8 μM and 19.9 μM or CD15- cells, respectively. Previous work has suggested that BKM120 has excellent blood brain barrier penetration [42]. Hence, we chose BKM120 for our *in vivo* studies. Current therapies for younger children with medulloblastoma have included the use of multiagent chemotherapeutic approaches including the chemotherapeutic agents, Temozolomide, cisplatinum [43, 44] and NVP-LDE225, a Smo antagonist developed by Novartis [45]. Hence, we examined the relative cytotoxicity of these drugs in CD15+ vs CD15- tumor cells. For these experiments, CD15+ and CD15- cells isolated from Smo *A1* Tg model were treated with BKM120, cisplatinum, TMZ, NVP-LDE225 or combination of BKM120 with cisplatinum or TMZ or NVP-LDE225. Interestingly, cisplatinum (IC_50_ 11.4 μM for CD15+ cells and 4.5 μM for CD15- cells) and TMZ (IC_50_ 30 μM for CD15+ cells and 20 μM for CD15- cells) has no effect, while NVP-LDE-225 (IC_50_ 2.8 μM for CD15+ cells and 2.5 μM for CD15- cells) has very less effect on survival of CD15+ cells (Fig 2B), suggesting that CSC/TPCs are resistant to conventional chemotherapy. S3A and S3B Fig shows dose dependent effect of cisplatin, and TMZ on CD15+ and CD15- cells. NVP-LDE-225 showed cytotoxic effects at high doses in CD15+ and CD15- cells, with no significant effect at 100 nM conc. (S3A & S3B Fig) Interestingly the combination of BKM120 & cisplatinum and BKM120 & TMZ did not result in augmentation of cytotoxicity activity in the CD15+ cells (Fig 2B). However, combination of BKM120 and NVP-LDE225 showed synergy in the CD15+ cells (Fig 2B). We next determined, if PI-3K inhibitors can block the elevated PI-3K signaling cascade in CD15+ TPCs. Treatment of CD15+ cells with different doses of BKM120 for 30 minutes, inhibited phosphorylation of AKT, its downstream target PRAS40 and mTOR substrates pS6, p4EBP1 in a dose-dependent manner (Fig 2C) exhibiting a dramatic decrease at 2.0 μM and almost complete inhibition at 10.0 μM. Real time PCR analysis demonstrated that 2 μM concentration of BKM120 completely suppressed the expression of SHH pathway genes viz., *gli1*, *gli2*, *N-Myc*, *C-Myc*, and *cyclin-D1* (Fig 2D).

### Differential sensitivity of CD15+ vs CD15- cells to PI-3 kinase inhibitor; Inhibition of the PI-3K pathway suppresses proliferation by inducing cell cycle arrest and inducing apoptosis in CD15+ TPCs but not in CD15- cells

**Fig 3:**
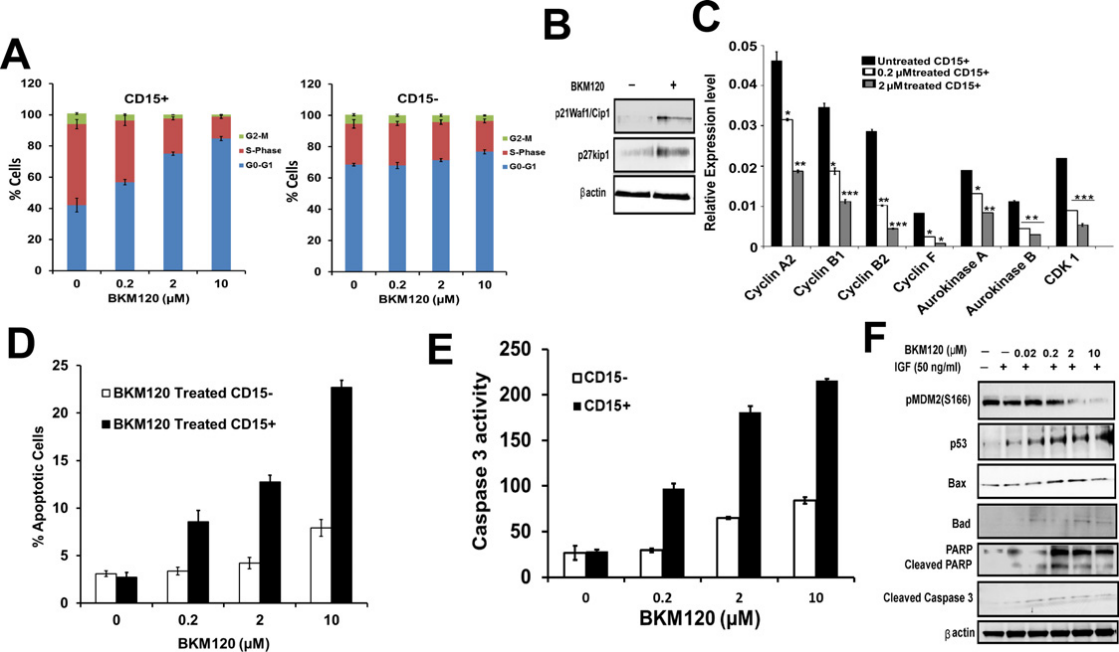
PI-3K inhibitors induce cell cycle arrest & apoptosis in CD15+ TPCs. (A) Fig shows cell cycle analysis data on BKM120 (0.2 μM, 2.0 μM and 10 μM) treated and untreated CD15+ and CD15- cells. Cells were harvested and analyzed for cell cycle profile (p < 0.05 for all phases compared to untreated group). (B) Western blot data for expression of p27^Kip1^ and p21^Waf1/Cip1^ in BKM120 (2.0 μM) treated CD15+ cells (30 min treatment). (C) Relative gene expression of cell cycle genes in BKM120 treated (0.2, 2.0 μM) CD15+ TPCs. Relative expression levels were normalized to GAPDH expression. (D) CD15+ and CD15- cells were treated with BKM120 (0.2 μM, 2.0 μM and 10 μM) for 24 hours, assayed for apoptosis by using the Annexin V ^FITC^ assay. (E) Increased caspase 3 activation with increasing conc. of BKM120. CD15+ and CD15- TPCs were treated with different conc. of BKM120 and used for fluorometric caspase 3 activity according to manufacturer’s instructions (Roche Diagnostic GmbH, Germany). (F) Western blotting of pMdm2 (S166), p53, Bax, Bad, cleaved caspase3 and cleaved PARP in CD15+ TPCs treated with different concentrations of BKM120. Results are mean ± SEM (n = 3–4 mice) for 3 independent experiments performed in triplicate (A, C, D & E). *P <0.05, **P <0.01 and ***P <0.001 vs. untreated, t test.

We next investigated if PI-3K inhibitors can induce cell cycle arrest in the CD15+ TPC compartment. For this, we first evaluated the baseline percentages of CD15+ and CD15- cells in different phases of cell cycle and found that 67% & 26% of the CD15- TPCs were in G0-G1 versus S phase respectively, while CD15+ cells comprise 48% and 45% of the cells in G0-G1 versus S phase, respectively (Fig 3A). Moreover treatment of CD15+ TPCs with BKM120 resulted in cell cycle arrest with a proportional increase in G0–G1 and a decrease in the number of cells in the S phase of the cell cycle (Fig 3A, left panel), while the treatment of CD15- cells with BKM120 showed no change in the percentage of cells in G0-G1 versus S phase of cell cycle (Fig 3A, right panel). The G1 arrest induced by BKM120 was correlated with the up-regulation of p27^Kip1^ and p21^cip1^ in BKM120 treated CD15+ TPCs (Fig 3B). Furthermore, the expression of cyclin A2, B1, B2, F, aurora kinase A, B, and CDK1 in CD15+ were completely suppressed by BKM120 at 2 μM concentration (Fig 3C). In order to determine if the cell cycle arrest phenotype was associated with the induction of apoptosis, Annexin-V FITC staining and caspase 3 activity assay were performed. Fig 3D reveals that BKM120 treated CD15+ TPCs induced a marked apoptotic response compared to untreated controls, while treatment of CD15- cell with BKM120 did not induce apoptosis (P >0.05). Consistent with this, fluorimetric caspase 3 enzyme assay showed that caspase 3 activity was increased in BKM120 treated CD15+ cells and not in CD15- cells (Fig 3E).

The AKT kinase is known to phosphorylate cytoplasmic MDM2 on serines 166 and 186, which promotes translocation of MDM2 from the cytoplasm into the nucleus where it mediates the degradation of p53 [46]. BKM120 was observed to potently block the phosphorylation of MDM2 at S166 in a dose-dependent manner with its complete inhibition at 10 μM concentration (Fig 3F). In addition, BKM120 also induced apoptosis in CD15+ TPCs by elevating the expression of pro-apoptotic protein p53 which was associated with the increased transcriptional up-regulation of downstream targets BAX and BAD (Fig 3F). BKM120 treatment increased levels of BAX in a dose dependent manner leading to caspase 3 activation as confirmed by Western blotting (Fig 3F).

### BKM120 inhibits tumor growth and preferentially blocks proliferation of CD15+ TPCs *in vivo*

**Fig 4:**
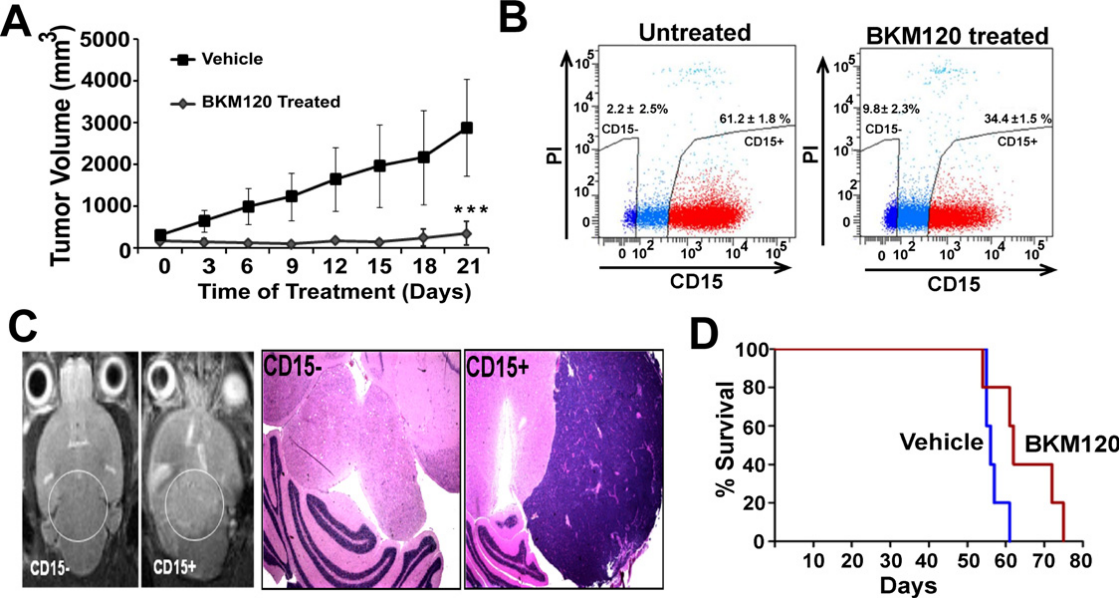
BKM120 inhibits tumor growth by depleting CD15+ TPCs *in vivo*. (A) Tumor growth was suppressed in BKM120 treated CD15+TPC xenografts. *Nu-nu* mice subcutaneously implanted with 10 million CD15+ TPCs were treated for 21 days with vehicle (control) or BKM120 at 30 mg/kg dose. Data are expressed as mean ± SEM (n = 6) (***P <0.001, t test) (B) FACS analysis on CD15+ cells isolated from BKM120 treated tumors as compared to untreated control mice. (C) Passaging of CD15+ or CD15- cells from second generation subcutaneous tumors into NOD SCID gamma (NSG) mice brain (orthotopic transplantation) *in vivo*. Left panel shows MRI images indicating, only CD15+ cell implanted NSG mice and not CD15- cells implanted mice formed tumor (white circle) in the MRI. Right panel shows histopathologic analysis of the CD15+ and CD15- transplanted NSG mice showing cortical tumor growth in only CD15+ implanted NSG mice. (D) 2 million CD15+ TPCs were intracranially transplanted into nude mice. After 40 days, mice displayed neurologic symptoms and were treated with vehicle (15% ethanol &15% cremophore, or BKM120 (30 mg/kg/day), 6 days a week for 21 days by oral gavage (*P <0.05 compared to vehicle, student’s t-test). Experiments were repeated 3 times with 7–8 mice in each group.

To address the functional relevance of up-regulated PI-3K signaling in TPC population, we asked whether BKM120 could block tumor growth in CD15+ TPCs xenografts grown subcutaneously in nude mice. As we expected, tumor growth was robust in the vehicle treated control group while growth was markedly suppressed (86% inhibition) in the BKM120 treated experimental group (Fig 4A). In order to characterize the antitumor effects of BKM120 *in vivo*, CD15+ and CD15- fractions were sorted from the treated and untreated tumors and used for apoptosis, cell cycle and proliferation studies. We observed that the proportion of CD15+ cells was much lower in the BKM120 treated group (45% reduction) (Fig 4B), indicating that treatment with PI-3K inhibitor BKM120 specifically depleted the CD15+ TPCs. These CD15+ TPCs isolated from BKM120 treated tumor showed less proliferation (S4A Fig) and increased apoptosis (S4B Fig) as compared to the vehicle treated controls. Furthermore, the expression of genes related to cell cycle and SHH pathways were also down-regulated in CD15+ TPCs isolated from BKM120 treated tumors as compared to vehicle treated controls (S4C & S4D Fig). Collectively, these results confirm the pharmacodynamic activity of BKM120 and indicate that BKM120 can function as a pro-apoptotic and an anti-proliferative agent for the CD15+ TPC compartment *in vivo*. In order to confirm our results that only CD15+ cells are capable of generating tumors, the CD15+ and CD15- cells isolated from the subcutaneous tumors were injected intracranially into NOD SCID mice. The MRI as well as H & E staining data clearly demonstrate that only CD15+ cells are capable of generating tumors (Fig 4C). Next, we evaluated the effect of BKM120 on the survival of mice, injected intracranially with CD15+ TPCs. For this, 2 million CD15+ TPC were injected into nude mice; after 40 days when mice displayed significant neurological symptoms c/w an advanced stage of intracranial tumor formation, they were divided into two groups. One group was treated with vehicle and another group was treated for 21 days with 30 mg/kg dose of BKM120. Fig 4D shows that BKM120 prolonged the survival of mice with advanced intracranial MB tumors (p < 0.05).

### Comparison of SmoA1 PTEN+/+ CD15+ TPC genomic signature to human MB subgroups

**Fig 5:**
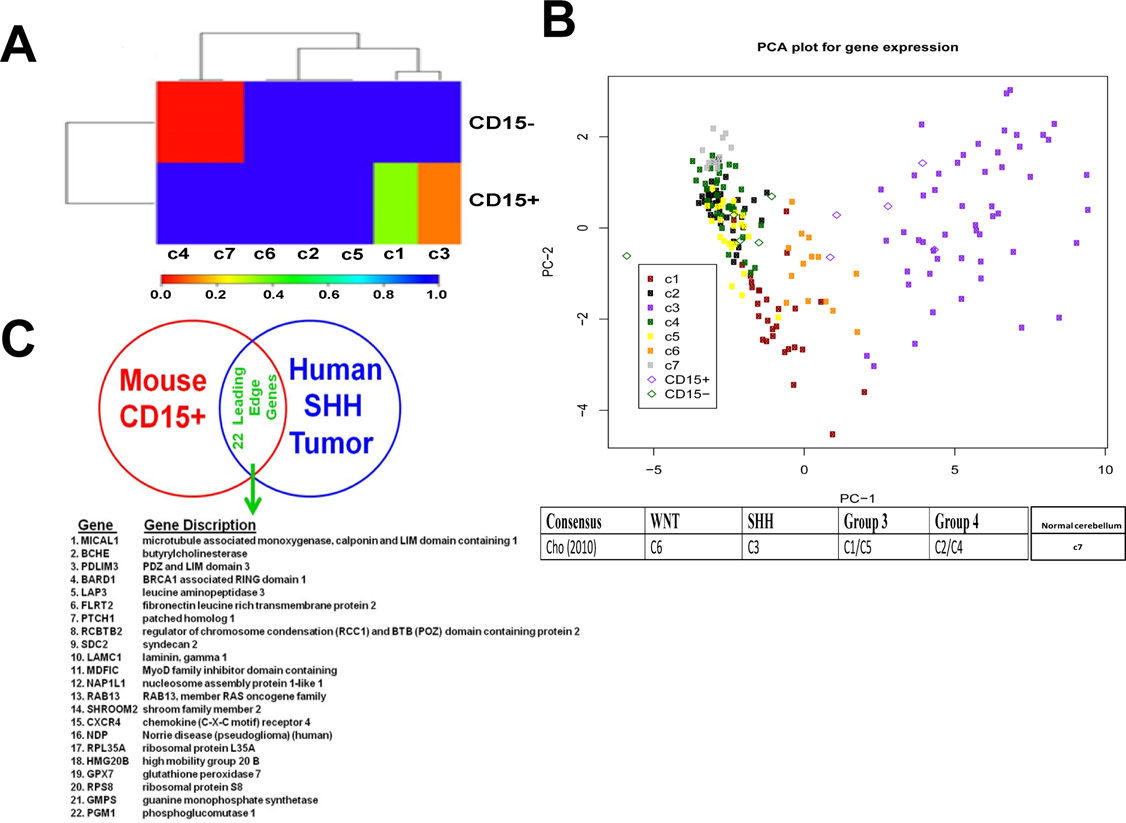
Similarity of CD15+ population from *SmoA1* PTEN +/+ tumors to the ‘c3/SHH’ subtype of human MB. (A) The heatmap shows the degree of similarity as quantified by the SubMap method [47] between gene expression levels of murine SmoA1Tg tumors (n = 5 paired CD15+ and CD15 samples) and 199 human tumors previously classified into 6 MB subtypes and normal control samples [4]. Each block in the heatmap corresponds to the p-value for similarity between the row category (mouse model samples, CD15+ or CD15-) and the column category (human tumor samples, classed by subtype). Blue suggests no similarity and red suggests strong similarity in gene expression levels. Note that the CD15- murine samples show the strongest similarity with human tumors subtypes c4 (Group 4) and c7 (normal CBL), while the CD15+ samples show similarity with c1 (Group 3) and c3 (SHH) subtypes. (B) Upper panel shows two dimensional Principal Components Analysis (PCA) plot comparing expression levels of the 22 leading edge genes from the SubMap analysis of Fig 5A. Data are from n = 5 paired CD15+ and CD15- murine SmoA1 PTEN+/+ tumors (open diamonds) and n = 199 human tumor samples (solid dots) previously classified into one of 6 MB subtypes [4] and reference normal samples. The human tumors are labeled according to subytpes as in Cho et al [4]. The first Principal Component, PC1, is plotted as the x-axis, and shows the major mode of variation in the data. PC1 is observed to separate the human tumors into three major groups, horizontally from right to left: c3 (purple, SHH subtype), c6 (orange, WNT subtype), and the combined group c1/c2/c4/c5/c7. Importantly, the CD15+ mouse samples (purple diamonds) are observed to associate with the human samples of SHH subtype (group c3), while the CD15- mouse samples (green diamonds) are clustered with the leftmost combined group c1/c2/c4/c5/c7 group containing normal human samples. The second PC, PC2 (y-axis) shows the next largest mode of variation in the data, and is seen to further divide the human the samples in the c1/c2/c4/c5/c7 combined group. The human normal samples (c7, grey) are clustered at the extreme high values of PC2, are largely distinct from the human tumor samples. Human subtypes c2 (black) and c4 (green) are highly overlapped as expected, but some separation is observed between human subtypes c1 (brown) and c5 (yellow), suggesting that these subtypes have distinct molecular phenotypes. Lower panel shows the major classification of medulloblastoma in 4 major subgroups by Taylor et al.[55] (C) List of 22 leading genes obtained from submap analysis.

To determine whether CD15+ population isolated from *SmoA1* PTEN+/+ tumors resembled human SHH-driven MB at a molecular level, we performed gene expression analysis on CD15+ and CD15- tumor cells isolated from *SmoA1* PTEN+/+ tumors and compared the resulting gene expression profiles with the gene expression profiles of recently identified 6 molecular subgroups of human medulloblastoma tumor samples (c1-c6) defined by Cho et al [4]. In this classification scheme, the c3 subgroup shows marked enrichment of genes associated with SHH signaling. Utilizing a subclass mapping algorithm [47] we generated a similarity metric between *SmoA1* PTEN+/+ tumors and the MB subgroups defined by Cho et al [4]. As expected in Fig 5, this analysis revealed a high degree of similarity between CD15+ population from *SmoA1* PTEN+/+ tumors and the ‘c3’ subtype of human MB, which is characterized by gene expression signatures indicative of SHH signaling pathway [4]. An unexpected finding was that the gene expression profile of the CD15- population correlates with ‘c7’ which represents gene expression in normal cerebellum (Fig 5A). To further verify that the CD15+ TPC population isolated from *SmoA1* PTEN+/+ tumors resemble ‘c3’ subtype of human MB, we used the leading edge analysis tool within GSEA [38] to identify and group related gene sets between the mouse and human genomes, i.e. those in which the significance is driven by an overlapping subset of genes (the "leading edge"). These results further verified that the CD15+ TPC population isolated from *SmoA1* PTEN+/+ resemble SHH driven c3 subtype of human medulloblastoma (S3 Table). Using the submap algorithm, we found 22 leading edge genes that putatively support the association between mouse CD15+ with SHH driven c3 subtype of human medulloblastoma. ComBat was used to remove the systematic variations between the two datasets and for generating the principal components analysis (PCA) plot. The plot shows that CD15+ and CD15- cells were readily distinguishable from one another (Fig 5B). From the PCA plot using the 22 leading edge genes (listed in Fig 5C) from the Submap analysis, we can see that the c3 subgroup is separated from the other subgroups, and that CD15+ associates with the c3 subgroup. Hence this plot graphically illustrates the association found in the Submap analysis. In contrast, comparison of CD15- gene expression signature aligns more with the remaining MB subgroups in this analysis. We extended our study to predict candidate drugs that might either repress or up regulate an expression signature, by using a publicly available resource Connectivity Map (CMAP). This resource is based on a reference collection of gene-expression profiles from cultured human cells treated with bioactive small molecules, together with pattern-matching software to mine these data [48]. Notably, analysis of human SHH-driven (Group c3) tumors using CMAP, suggested that genes regulated by PI-3K/mTOR (LY294002, Sirolimus) and MAPK/MEK (U0125) inhibitors are also enriched in these tumors (S4 Table), which is in close agreement with our results showing the elevated expression of these pathways in CD15+ TPCs. Furthermore this analysis revealed the identification of many other compounds predicted to repress the SHH driven tumors (S4 Table). The list included several compounds that are either approved or undergoing clinical trials in different cancer types. Several drug classes which are listed top on the list include topoisomerase inhibitors (camptothecin, ellipticine, doxorubicin, etoposide, thioguanine, thioguanosine), multiple histone deacetylase inhibitors (trichostatin A, vorinostat, 5162773, 5186223), PARP inhibitors (1,5-isoquinolinediol, 3-aminobenzamide, phenanthridinone), proteasome inhibitors (MG132, 1,4 chrysenequinone) and cyclin-dependent kinases (CDKs) inhibitors (alsterpaullone, 02974170002B, GW-8510).

### Blocking the PI-3K signaling pathway inhibits proliferation of CD15+ TPC in primary human medulloblastoma and in a MBSHH PDX model

**Fig 6:**
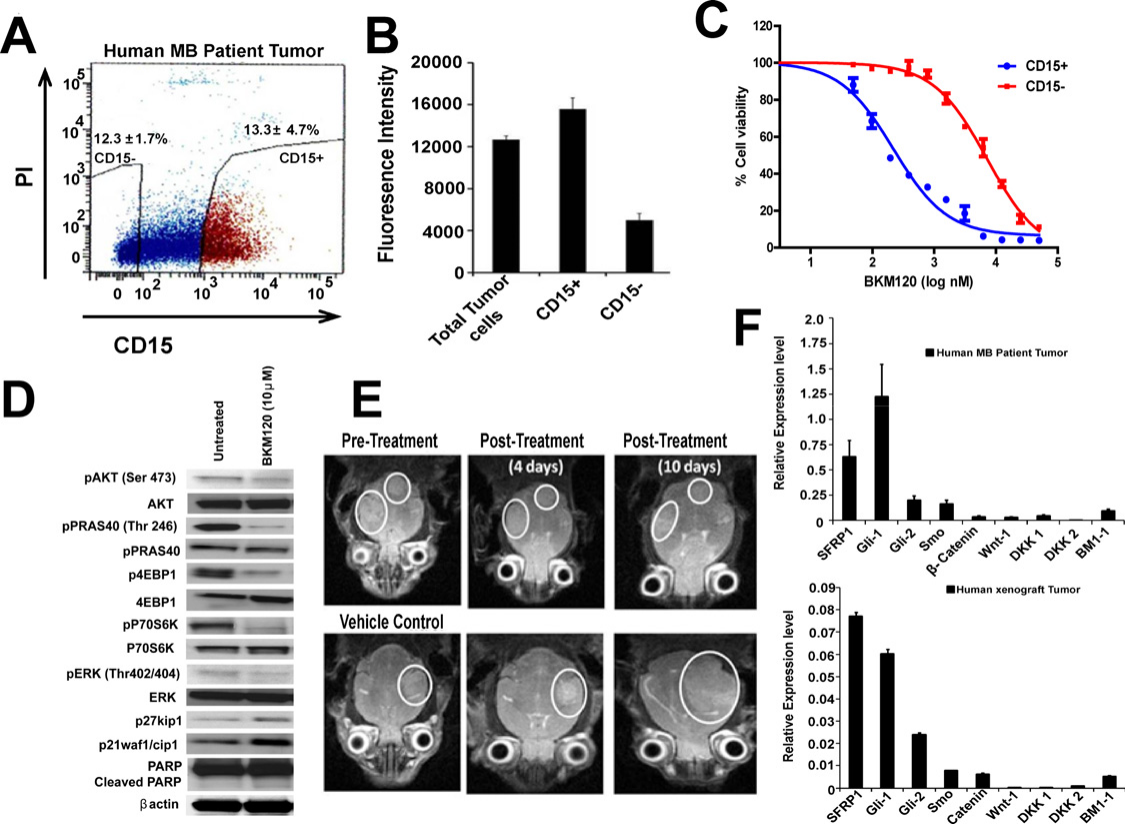
Augmented antitumor activity of BKM120 against human CD15+ cells isolated from SHH MB PDX l. *(*A) FACS analysis of CD15 expression in a human SHH subgroup medulloblastoma tumor. (B), Total tumor cells, FACS sorted CD15+ and CD15− tumor cells were used for cell viability assay in presence of BKM120 using AlamarBlue®. (C) CD15+ and CD15- TPCs from human SHH MB patient tumor were cultured in serum-free media containing no additive, DMSO (vehicle), BKM 120 at different conc. followed by cell viability assay using AlamarBlue®. (D) Western blot analysis demonstrates suppression of PI-3K signaling in BKM120 treated CD15+ TPCs isolated from a human medulloblastoma tumor. (E) BKM120 treatment significantly prolonged survival of mice and reduce the tumor size. 2 million CD15+ TPCs were transplanted intracranially into *nu/nu* mice. After 90 days, MRIs taken in the coronal plane show the formation of a tumor (white circle) in nude mice followed by separation of mice into two random groups. Mice in group1 were treated with vehicle (15% ethanol &15% Cremophore) and those in group 2 were treated with BKM120 (30 mg/kg/day), 5 days / week for 14 days by oral gavage. (F) Sub-classification of human MB tumor and PDX by real time RT-PCR. SHH pathway genes (SFRP1, gli-1, gli-2 and Smo) are up-regulated in human MB (upper panel) and xenograft (lower panel) as compared to WNT pathway genes (Wnt-1, DKK1, DKK2) and non SHH/non Wnt (BMI-1), validated by real time RTPCR.

Finally, we validated our hypothesis by testing these PI-3K inhibitors in primary human medulloblastoma tumor cells and a patient-derived xenograft (PDX) SHH MB model. For this, we first analyzed the primary human MB tumor and PDX by H & E staining and by RT PCR analysis of stem cell marker genes (S5A and S5B Fig).These results and previous report [49] suggest that this MB PDX is a SHH subgroup tumor. Histopathologic diagnosis of MB was confirmed by attending pediatric pathologist at Rady Children’s Hospital. MB tumor cells were isolated and evaluated using FACS analysis for CD15 expression (Fig 6A). In order to evaluate potential contamination of CD15+ neutrophils in our experiments using human medulloblastoma PDX, we performed FACS analysis using CD15 and CD66 antibodies. An almost undetectable level of human neutrophils (< .04% of total CD15+ population) was detected (data not shown). We evaluated the expression of stem cell markers in the tumor cells isolated from PDX and found significantly higher expression of oct4, sox2, nanog, klf4, cxcr4, musashi, CD133 and ngfr in patient samples when compared to normal cerebellum which served as a control (S5B Fig). Most notably, the expression of Pten is lower in the tumor cells isolated from this PDX as compared to control. A further characterization of the CD15+ population from the PDX reveals TPC properties and a higher proliferative capacity (Fig 6B, S5C Fig). Moreover, BKM120 potently inhibit the proliferation of the CD15+ TPC population by 21 fold, while there is minimal effect on CD15- population (IC_50_ for CD15+ cells is 0.218 μM and for CD15- cells it is 7.178 μM) (Fig 6C). Therefore, we investigated if exposure of cytotoxic agent, cisplatinum, NVP-LDE-225 and TMZ has a similar effect on the CD15+ TPC population isolated from PDX. Interestingly, cisplatinum and TMZ has no effect, while NVP-LDE-225 has very minimal effect on proliferation of CD15+ cells isolated from PDX (S5D Fig). Furthermore, BKM120 at 10 μM concentration completely blocked the phosphorylation of AKT, and its downstream targets, PRAS40 and mTOR substrates pS6, p4EBP1 in human MB TPCs (Fig 6D). The higher expression of p27 and p21 protein levels and cleavage of PARP upon treatment with BKM120 suggests that BKM120 increase cell cycle arrest and induce apoptosis in CD15+ cells isolated from human medulloblastoma (Fig 6D). Overall, these results suggest that the inhibition of PI-3K will be potently inhibitory for TPC survival *in vivo*. We confirm the *in vivo* efficacy of BKM120 in the PDX and we observed that this inhibitor blocked tumor growth and enhance survival of mice as shown in MRI images (Fig 6E). Finally, we subclassified the tumor obtained from the primary tumor sample as well as from PDX specimen for the expression of specific marker genes restricted to SHH, Wnt, Non SHH/Non Wnt pathway. We found that SHH pathway genes are similarly upregulated in the primary tumor as well as in PDX (Fig 6F). Importantly, these analyses were completed within 3–4 months in “real time” while patient received standard of care for MB which includes chemotherapy and radiotherapy which is associated with 30 percent recurrence rate.

## Discussion

The PI-3K/AKT pathway has been shown to be important for the proliferation of TPCs in both solid tumors and leukemias [31–33]. The role of PTEN-PI-3K/AKT pathway in the pathogenesis and tumorigenicity of medulloblastoma has also been extensively studied in bulk tumor [14–16, 50, 51]. Herein, we report that, PI-3K signaling is highly elevated in CD15+ TPCs isolated from Smo *A1*Tg model of medulloblastoma and is required for the proliferation of TPCs. PI-3K inhibitors exert a preferential effect on CD15+ TPCs isolated from Smo *A1*Tg model of medulloblastoma and in human patient derived xenografts, with minimal to no effect on CD15- population. In contrast, the cytotoxic chemotherapeutic agent, cisplatinum, TMZ and SHH inhibitor, NVP-LDE-225 do not display *in vitro* cytotoxicity against CD15+ TPCs isolated from this mouse model or a SHH subgrouped human patient derived MB xenograft. In contrast, BKM120 as a single agent, blocked the proliferation of CD15+ TPCs with minimal effect on CD15- cells. Most notably BKM120, was observed to: **1**) induce p21waf1, p27kip1 and p53 expression **2**) suppress proliferation and the expression of proliferative markers, cyclin D, MycN, gli-1, gli-2 **3)** induce apoptosis in the CD15+ TPC compartment and in the SHH subgroup of patient derived tumor cells (PDX) and **4)** suppress tumorigenesis and increase host survival in an *in vivo* CD15+ TPC xenograft model.

Various clinical reports suggest that SHH- driven medulloblastoma patients treated with Smo antagonists, initially show dramatic tumor regression followed by rapid tumor recurrence [12, 13]. Buanamici *et al* has reported the upregulated PI-3K signaling as one of the potential mechanism of resistance developed in SHH driven medulloblastoma [14]. They reported that BKM120 in combination with Smo antagonist LED225 showed delayed tumor growth in Patch+/−p53-/- mouse model [14]. In our experiments, BKM120 treatment caused a substantial tumor reduction and most notably it suppressed the percent of CD15+ cells in the subcutaneous tumor, by inducing them to undergo apoptosis. Our findings have implications for the clinical development of PI-3K inhibitors including BKM120 in the treatment of SHH driven medulloblastoma. The observation that CD15+ TPC exhibited 10- to 20- fold greater sensitivity to PI-3K inhibitors than CD15-TPCs (Fig 2A) suggests that clinical trials designed with TPC-directed endpoints may facilitate demonstration of efficacy at sub-MTD doses. Current first-line chemotherapy generally consists of cytotoxic agents, such as platinum agents (cisplatinum) and etoposide. NVP-LDE-225, another drug we used in our study is in Phase II clinical trials for patients with hedgehog pathway activated relapsed medulloblastoma. While these agents may effectively debulk tumors and control disease initially, tumors invariably recur due to ineffective control of TPC. In the present study, we observed that the CD15+ TPC isolated from Smo *A1* mouse model as well as human PDX are markedly resistant to cisplatinum, TMZ and NVP-LDE-225 whereas CD15- population is more sensitive to these agent. Surprisingly, no synergy was noted when BKM120 was combined with cisplatinum and TMZ suggesting independent mechanisms for cellular cytotoxicity of cisplatinum in CD15- cells. It is interesting to speculate that CD15+ cells will arise in humans following treatment with cisplatinum chemotherapy due to this resistance pattern and that this population of cells would be sensitive to BKM120 treatment in sequence. Hence, treating SHH driven MB with TPC-targeting agent viz. PI-3K inhibitors is expected to block CD15+ TPC mediated tumor recurrence observed in MB if combined with standard of care agents. Our result that the CD15+ TPC population display stem cell markers and form large robust neurospheres *in vitro* suggest that PI-3K inhibitors preferentially target the TPC/cancer stem cell compartment. In agreement with our study, recent study has shown that PI-3K/mTOR inhibitor VS5584 preferentially target the aldefluor positive cancer stem cell compartment [34].

In conclusion, we have identified a role for PI-3K signaling in the proliferation and survival of TPC dependent c3 SHH subtype of MB. Our results provide the first evidence that PI-3K inhibitors have cancer stem cell disease modifying activity *in vitro* and *in vivo*. We expect that these findings will positively impact on our understanding of the signaling pathways operational in the cancer stem cell which promotes its tumorigenicity, survival and resistance. Bioinformatic analysis comparisons of the genomic signature of the CD15+ TPC population isolated from murine tumors reveals a similarity with the c3 subgroup of SHH driven human MB tumors. These results suggest that the research conducted on murine SmoA1Tg model will be potentially applicable in SHH driven human MB patients. Recent report by Pei et al also used the similar methodology to compare the data from murine Myc MB model to expression profiles from a distinct set of human samples [52]. Consistent with this, there are other reports which validate the use of this methodology to compare murine data with human samples [53, 54]. An important distinction between these reports and our work relates to our focus to compare the gene expression pattern within the CD15+ TPC cells to the different MB subgroups and to ultimately compare effects of genetic loss in PTEN using these methods on the TPC cell phenotype in an effort to discover resistance mechanisms to PI-3 kinase inhibitory regimens. There are no reports in the literature comparing mRNA gene expression in CD15+TPCs vs CD15- nonTPCs and/or comparing the CD15+ TPC signature to the human medulloblastoma tumor mRNA expression in subgroups of MB. Taken together, our study provides new avenues to perform genomic manipulations and multiple ‘omic’ analyses on the drug treated murine CD15+ TPC to determine possible mechanisms of resistance in the TPC compartment and thereby discover more efficacious treatments for this and other cancer stem cell driven diseases. Given the paucity of gain of function mutations in PIK3CA or loss of function mutations in PTEN observed in medulloblastoma tumors as determined by whole genome sequencing (1–3%), we envision the application of PI-3K inhibitors as adjuncts to existing chemotherapy and radiotherapy regimens in the future treatment of MB once Phase I trials are completed in pediatric oncology. Finally, we performed within 1–3 months of diagnosis *in vivo* molecular profiling, synthetic lethality and drug sensitivity screening of a primary highly anaplastic MB tumor (Fig 6) and its corresponding PDX in “real time” as a potential “clinical proof of concept” for the application of personalized pediatric oncology in a high risk disease setting.

## Supporting Information

S1 Fig *SmoA1* tumors are propagated by CD15+ cells. (A) FACS data showing the isolation of pure population of CD15+ cells from *Smo A1* tumors. (B) CD15+ and CD15- cells were implanted intracranially into nude/*nu-nu* mice. Upper panel shows H&E staining of Secondary tumor from a *nu-nu* host that received 2 × 10^6^ CD15+ (Left panel) and CD15- cells (Right panel). Small box in Upper right panel shows H&E at 20X. Lower panel shows Ki67 staining of same tumor. Scale bar = 200 μm. (C) Relative gene expression of stem cell markers in the CD15+ and CD15- population isolated from *SmoA1* tumors. Graphs represent mean ± SEM. Statistical significance is assessed by two sample *t*-test where *denotes *P*<0.05, ** denotes *P*<0.01 and *** denotes *P*<0.001.
(TIF)

S2 Fig CD15+ cells from *SmoA1* tumors have a distinct expression profile with increased proliferation and cell survival capacity. (A) Figure shows relative expression levels of genes related to proliferation and cell survival in CD15+ vs. CD15- population isolated from *SmoA1* tumors (n = 3). (B) Heat map showing activation of SHH pathway genes in CD15+ cells (n = 7) compared to CD15- (n = 5). Colors illustrate fold changes, Red: up-regulation; green: down-regulation; black: no change. The bar code on the bottom represents the color scale of the log 2 values. (C) Left panel shows validation of differential gene expression for SHH pathway genes in CD15+ vs. CD15- population by RTPCR. Right panel shows Western blot revealing high expression of gli1, gli2 and cyclin D1. (D) Heat map showing activation of genes related to angiogenesis in CD15+ cells (n = 7) compared to CD15- (n = 5). Data are representative of three independent experiments. Values are mean ± SEM (*n* = 6–8) (A & C). Statistical significance is assessed by two sample *t*-test where *denotes *P*<0.05, ** denotes *P*<0.01 and *** denotes *P*<0.001.
(TIF)

S3 Fig **Dose dependent effect of Cisplatin, NVP-LDE-225 and TMZ on CD15+ and CD15- cells isolated form Smo A1Tg mice** (A & B) CD15+ and CD15- cells were treated with different conc. of Cisplatin, NVP-LDE-225 and TMZ (0.1 μM, 1.0 μM and 10.0 μM). After 48 hr, AlamarBlue® was added and plates were incubated at 37°C in 5% CO_2_ for 6 hours. Fluorescence signals were read as emission at 590 nm after excitation at 560 nm.
(TIF)

S4 Fig BKM120 suppresses tumor growth by increasing apoptosis of CD15+ TPC and down regulating the expression of cell cycle and SHH pathway genes. (A) AlamarBlue data validating the less proliferation tendency of CD15+ cells derived from BKM120 treated tumors as compared to untreated ones. (B) BKM120 reduces cell proliferation in CD15+ CSC by inducing apoptosis. CD15+ and CD15- cells isolated from BKM120 treated and untreated tumors were assayed for apoptosis by using the Annexin V ^FITC^ assay. (p = 0.06). (C & D) BKM120 suppresses tumor growth by targeting cell cycle and SHH genes in CD15+CSC population. Expression of cell cycle genes (C) and SHH genes (D) in CD15+ cells derived from BKM120 treated and untreated subcutaneous tumors. Expression data is normalized to GAPDH. Values are mean ± SEM (*n* = 6–8) (A-D). Statistical significance is assessed by two sample *t*-test where *denotes *P*<0.05, ** denotes *P*<0.01 and *** denotes *P*<0.001.
(TIF)

S5 Fig Characterization of CD15+ isolated from medulloblastoma tumor patient sample. (A) A small portion of the patient tumor was fixed in formalin, paraffin embedded and used for H & E staining. (B) Relative gene expression of stem cell markers in the tumor cells isolated from PDX. RNA isolated from normal cerebellum was used as a control. (C) *In-vitro* cell proliferation of total tumor cells, CD15+ and CD15- cells obtained from patient tumor. Total tumor cells and FACS sorted CD15+ CSCs have the ability to form neurospheres in the culture. Cells are cryopreserved and evaluated for sensitivity against kinome panel and siRNA screens for patient specific synthetic lethality effects in combination with PI-3K inhibitors. (D) CD15+ cells isolated from PDX were treated with different conc. of cisplatin (Left panel). Right panel shows the cell viability of CD15+ cells treated with 100nM conc. of cisplatinum, TMZ, NVP-LDE-225 either alone or in combination with BKM 120.
(TIF)

S1 Table List of primer sequence used in Real Time PCR analysis. (DOC)

S2 Table Differentially expressed genes of different pathways analyzed by microarray data in CD15-vs. CD15+ FACS sorted population from *SmoA1* x PTEN+/+ tumors. (XLS)

S3 Table Table shows leading edge genes that were used to perform PCA analysis and examine the similarity between the human MB sub classified tumors and CD15+ CSCs derived from *SmoA1* PTEN+/+ Mouse MB. (XLS)

S4 Table Connectivity map analysis: Table shows compounds whose gene expression signatures closely match those of human Group c3 tumors. Among the top 50 compounds are several PI3K, MAPK/MEK and mTOR inhibitors (highlighted). These results are consistent with analysis of murine SHH tumors, which suggests activation of the PI3K/mTOR & MAPK/MEK pathway.
(XLS)

## Abbreviations

CSC: cancer stem cell
TPC: tumor propagating cell
PI-3K: phosphoinositide 3-kinase
PTEN: phosphatase and tensin homolog
PDX: patient-derived xenograft
