# Supplementary figures and images for: PI-3K Inhibitors Preferentially Target CD15+ Cancer Stem Cell Population in SHH Driven Medulloblastoma
Source: PLoS One. 2016 Mar 3;11(3):e0150836. doi: 10.1371/journal.pone.0150836 (PMC4777592; doi:10.1371/journal.pone.0150836)

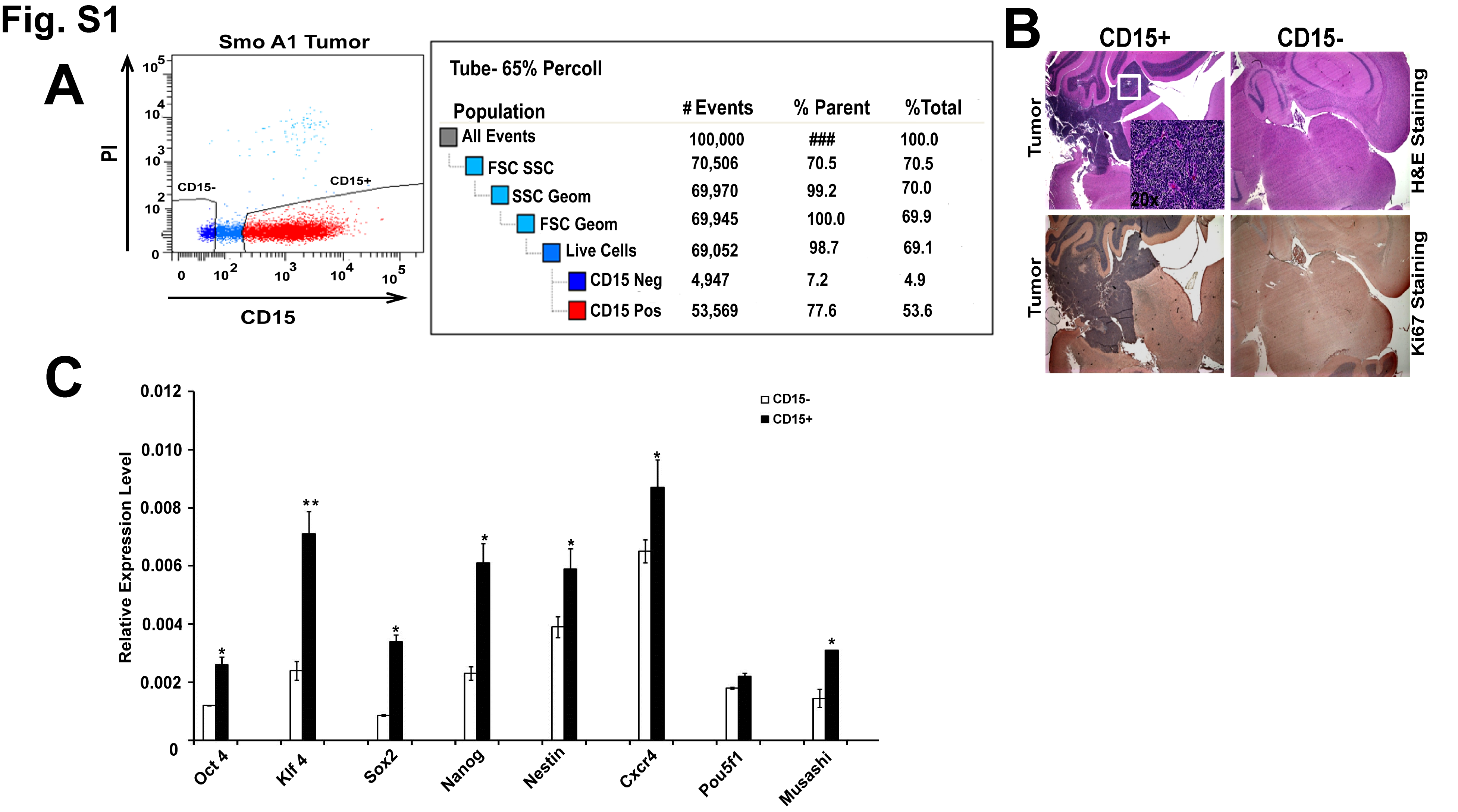

Supplement: S1 Fig — (A) FACS data showing the isolation of pure population of CD15+ cells from Smo A1 tumors. (B) CD15+ and CD15- cells were implanted intracranially into nude/nu-nu mice. Upper panel shows H&E staining of Secondary tumor from a nu-nu host that received 2 × 10^6^ CD15+ (Left panel) and CD15- cells (Right panel). Small box in Upper right panel shows H&E at 20X. Lower panel shows Ki67 staining of same tumor. Scale bar = 200 μm. (C) Relative gene expression of stem cell markers in the CD15+ and CD15- population isolated from SmoA1 tumors. Graphs represent mean ± SEM. Statistical significance is assessed by two sample t-test where *denotes P<0.05, ** denotes P<0.01 and *** denotes P<0.001. (TIF) [file pone.0150836.s001.tif]

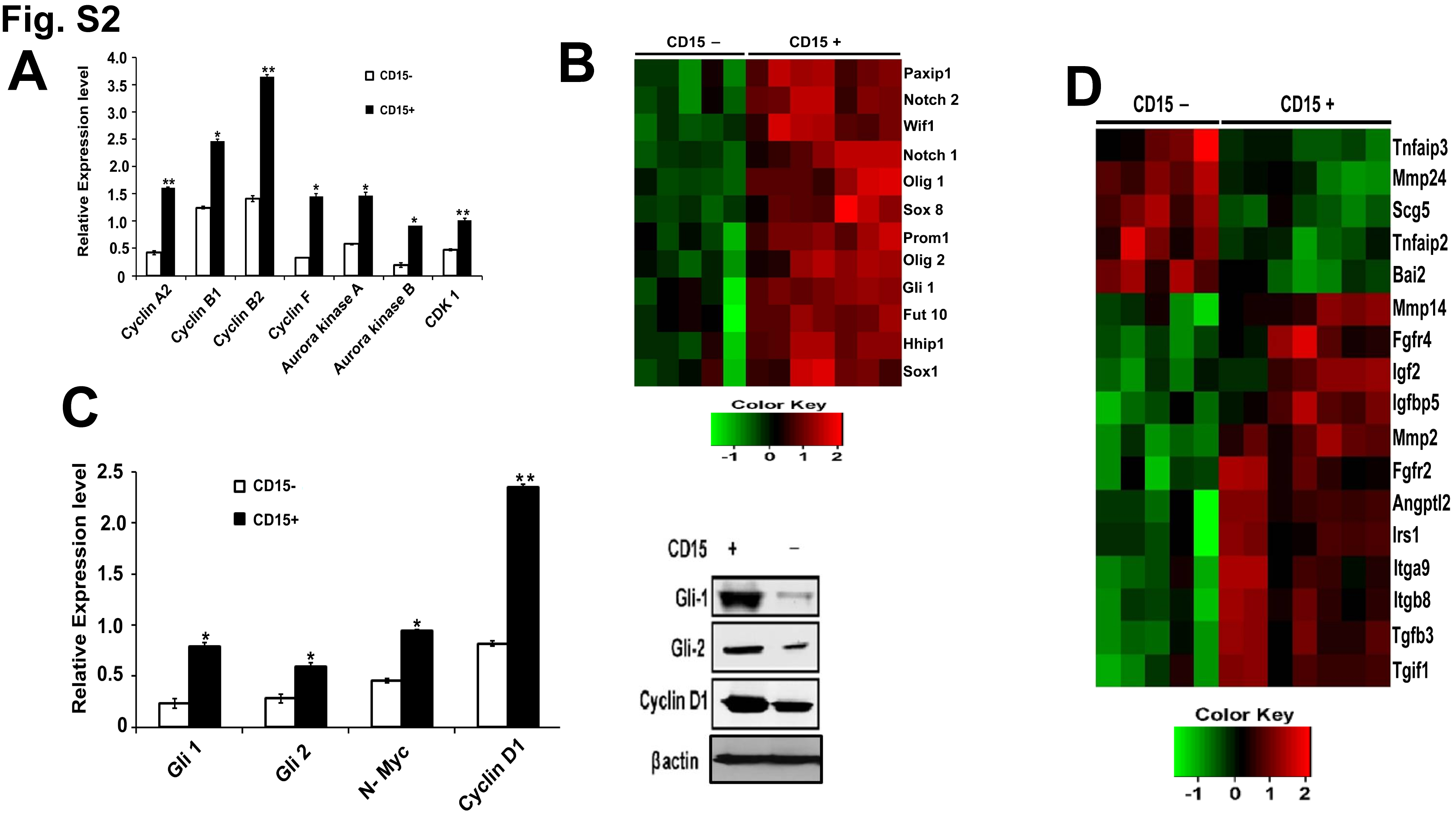

Supplement: S2 Fig — (A) Figure shows relative expression levels of genes related to proliferation and cell survival in CD15+ vs. CD15- population isolated from SmoA1 tumors (n = 3). (B) Heat map showing activation of SHH pathway genes in CD15+ cells (n = 7) compared to CD15- (n = 5). Colors illustrate fold changes, Red: up-regulation; green: down-regulation; black: no change. The bar code on the bottom represents the color scale of the log 2 values. (C) Left panel shows validation of differential gene expression for SHH pathway genes in CD15+ vs. CD15- population by RTPCR. Right panel shows Western blot revealing high expression of gli1, gli2 and cyclin D1. (D) Heat map showing activation of genes related to angiogenesis in CD15+ cells (n = 7) compared to CD15- (n = 5). Data are representative of three independent experiments. Values are mean ± SEM (n = 6–8) (A & C). Statistical significance is assessed by two sample t-test where *denotes P<0.05, ** denotes P<0.01 and *** denotes P<0.001. (TIF) [file pone.0150836.s002.tif]

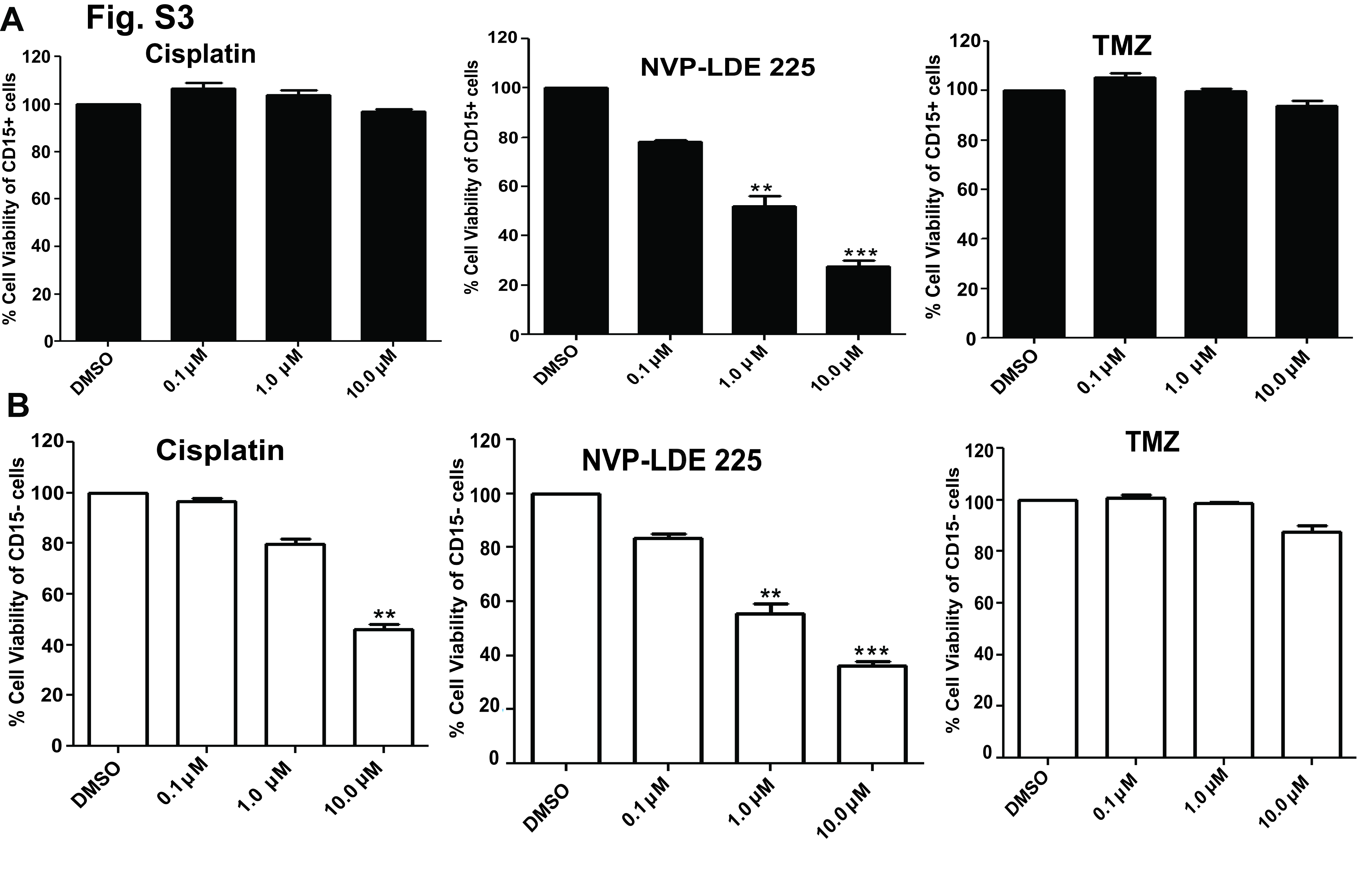

Supplement: S3 Fig — Dose dependent effect of Cisplatin, NVP-LDE-225 and TMZ on CD15+ and CD15- cells isolated form Smo A1Tg mice (A & B) CD15+ and CD15- cells were treated with different conc. of Cisplatin, NVP-LDE-225 and TMZ (0.1 μM, 1.0 μM and 10.0 μM). After 48 hr, AlamarBlue® was added and plates were incubated at 37°C in 5% CO2 for 6 hours. Fluorescence signals were read as emission at 590 nm after excitation at 560 nm. (TIF) [file pone.0150836.s003.tif]

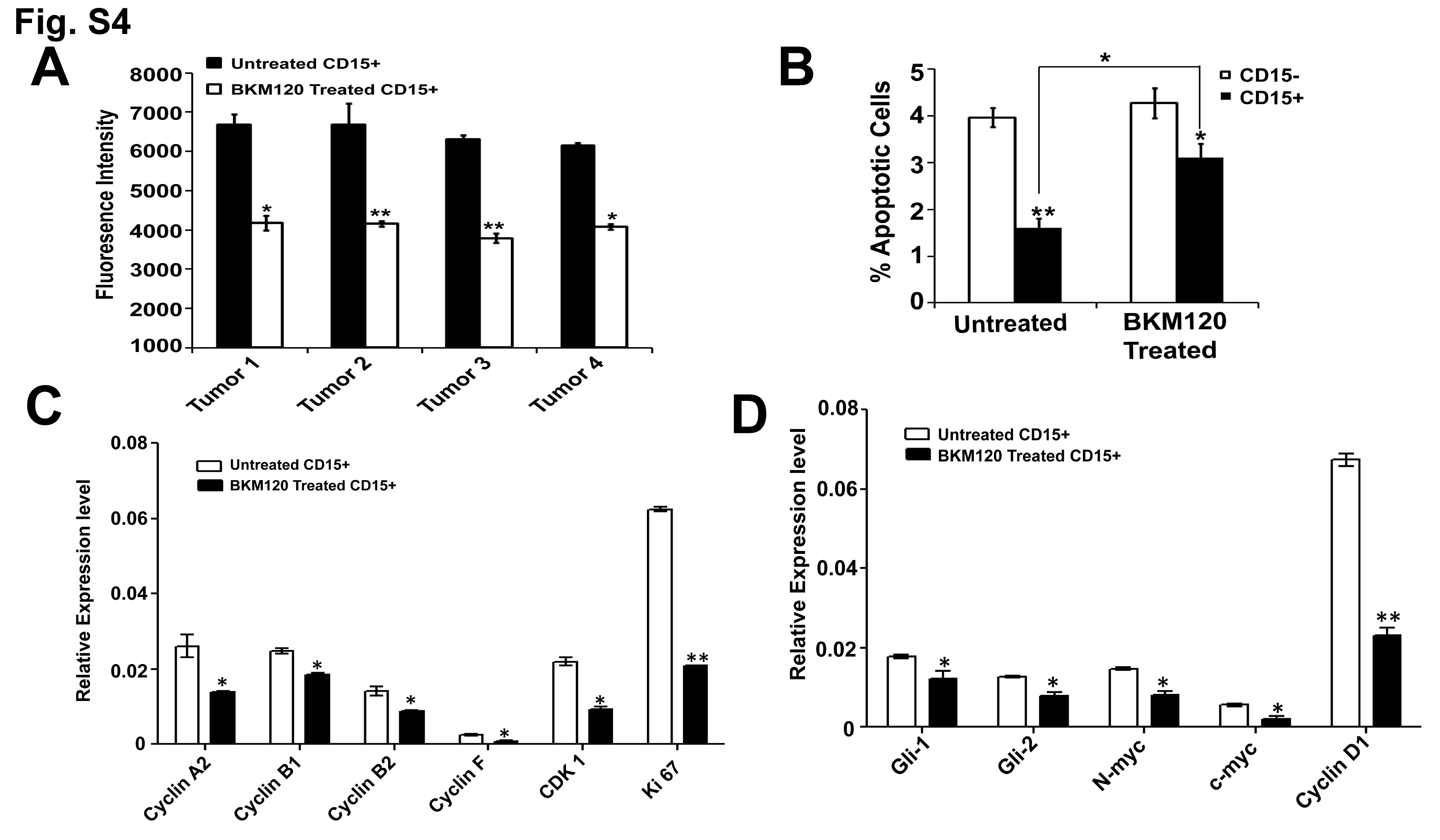

Supplement: S4 Fig — (A) AlamarBlue data validating the less proliferation tendency of CD15+ cells derived from BKM120 treated tumors as compared to untreated ones. (B) BKM120 reduces cell proliferation in CD15+ CSC by inducing apoptosis. CD15+ and CD15- cells isolated from BKM120 treated and untreated tumors were assayed for apoptosis by using the Annexin V FITC assay. (p = 0.06). (C & D) BKM120 suppresses tumor growth by targeting cell cycle and SHH genes in CD15+CSC population. Expression of cell cycle genes (C) and SHH genes (D) in CD15+ cells derived from BKM120 treated and untreated subcutaneous tumors. Expression data is normalized to GAPDH. Values are mean ± SEM (n = 6–8) (A-D). Statistical significance is assessed by two sample t-test where *denotes P<0.05, ** denotes P<0.01 and *** denotes P<0.001. (TIF) [file pone.0150836.s004.tif]

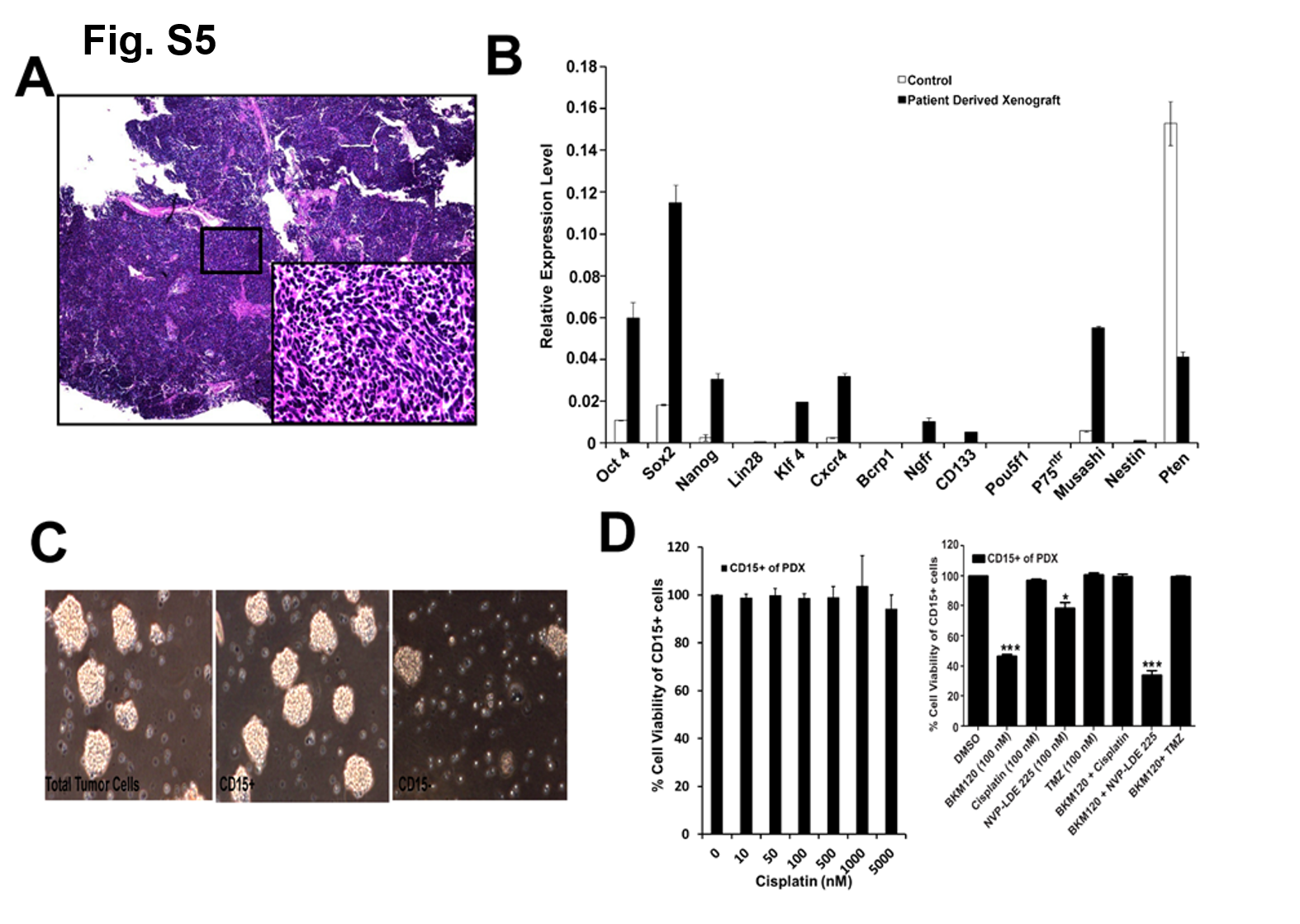

Supplement: S5 Fig — (A) A small portion of the patient tumor was fixed in formalin, paraffin embedded and used for H & E staining. (B) Relative gene expression of stem cell markers in the tumor cells isolated from PDX. RNA isolated from normal cerebellum was used as a control. (C) In-vitro cell proliferation of total tumor cells, CD15+ and CD15- cells obtained from patient tumor. Total tumor cells and FACS sorted CD15+ CSCs have the ability to form neurospheres in the culture. Cells are cryopreserved and evaluated for sensitivity against kinome panel and siRNA screens for patient specific synthetic lethality effects in combination with PI-3K inhibitors. (D) CD15+ cells isolated from PDX were treated with different conc. of cisplatin (Left panel). Right panel shows the cell viability of CD15+ cells treated with 100nM conc. of cisplatinum, TMZ, NVP-LDE-225 either alone or in combination with BKM 120. (TIF) [file pone.0150836.s005.tif]
