# Supplementary material for: PI-3K Inhibitors Preferentially Target CD15+ Cancer Stem Cell Population in SHH Driven Medulloblastoma
Source: PLoS One. 2016 Mar 3;11(3):e0150836. doi: 10.1371/journal.pone.0150836 (PMC4777592; doi:10.1371/journal.pone.0150836)
Supplement: S1 Table — (DOC) [file pone.0150836.s006.doc]

**q-Real Time RT PCR Primers of mouse cell cycle genes**

1. mCyclin A2

F ACATTCACACGTACCTTAGGGA

R CATAGCAGCCGTGCCTACA

1. mCyclin B1

F AAGGTGCCTGTGTGTGAACC

R GTCAGCCCCATCATCTGCG

1. mCyclin B2

F GCCAAGAGCCATGTGACTATC

**R** CAGAGCTGGTACTTTGGTGTTC

1. mCyclin F

F GAAGGACTTTACAAGCCTGTGT

R CCAGGGCGGAAATGATCTCC

1. mAurorakinase A

F CTGGATGCTGCAAACGGATAG

R CGAAGGGAACAGTGGTCTTAACA

1. mAurokinase B

F CAGAAGGAGAACGCCTACCC

R GAGAGCAAGCGCAGATGTC

1. mCDK 1

F AGAAGGTACTTACGGTGTGGT

R GAGAGATTTCCCGAATTGCAGT

1. mKi-67

F TTTGAGGCACGCCTGATCC

R GGAGACGTGAGACGAGTCCAT

**q-Real Time RT-PCR of mouse hedgehog target genes**

1. mGli1

F ATGGAGAGAGCCCGCTTCTTT

R TTATGGAGCAGCCAGAGAGACCAG

1. mGli2

F CAACGCCTACTCTCCCAGAC

R GAGCCTTGATGTACTGTACCAC

1. mCyclin D1

F ACCCTGACACCAATCTCCTCAAC

R TGGATGGCACAATCTCCTTCTG

1. mN-MYC

F AACAAGGCGGTAACCACTTTCAC

R TGCTGCTGATGGATGGGAAC

1. mMYC,

F TCAAGAGGCGAACACACAAC

R GGCCTTTTCATTGTTTTCCA

**q-Real Time RT-PCR primers for Sub-classification of human MB tumor and PDX**

1. hSFRP1

F TCTACTGGCCCGAGATGCTTAAG

R CACAGGGAGGACACACCGTTGTGC

1. hGLi-1

F CCCAAGGCTCTAGGTGGAAC

R CTGGGGGTAATGGGAAAAGA

1. hGLi-2

F CAGCATCTCTTGCCACCATT

R AAGCCGGATCAAGGAGATGT

1. hSMO

F TCTACGTCAATGCGTGCTTC

R CACAAACCAAACCACACCAG

1. **hβ-catenin**

F TTCGCCTTCACTATGGACTACC

R GCACGAACAAGCAACTGAACTA

1. **h Wnt1**

F CGACCTCGTCTACTTCGAG

R ACCAGTGGAAGGTGCAGTTG

1. DKK-1

F GGAATAAGTACCAGACCATTGACAAC

R GGGACTAGCGCAGTACTCATCA

1. DKK-2

F CCCAGTACCCGCTGCAATAATGGC

R CTGTGCCGAGTACCATCCAG

1. BMI-1

F GGAGGAGGTGAATGATAAAAGAT

R AGGTTCCTCCTCATACATGACA

**q-Real Time RT-PCR of mouse stemness marker genes**

1. **mOct4**

F ACATCGCCAATCAGCTTGG

R AGAACCATACTCGAACCACATCC

1. **mKlf4**

F GCACACCTGCGAACTCACAC

R CCGTCCCAGTCACAGTGGTAA

1. **mSox2**

F ACAGATGCAACCGATGCACC

R TGGAGTTGTACTGCAGGGCG

1. **mNanog**

F GAAATCCCTTCCCTCGCCATC

R CTCAGTAGCAGACCCTTGTAAGC

1. **mNESTIN**

F TGAGGGTCAGGTGGTTCTG

R AGAGCAGGGAGGGACATTC

1. **mCXCR4**

F TCAGTGGCTGACCTCCTCTT

R CTTGGCCTTTGACTGTTGGT

1. **mPou5f1**

F ACGAGTGGAAAGCAACTCA

R AGATGGTGGTCTGGCTGAAC

1. **Mmusashi-1 (Msi1)**

F TAG TTC GAG GGA CAG GCT CT

R GTT GAG GGA CAG GCA GTA GC

1. **mPTEN**

F AATTCCCAGTCAGAGGCGCTATGT

R GATTGCAAGTTCCGCCACTGAA CA

1. **m GAPDH**

F TTCACCACCATGGAGAAGGC

R CCCTTTTGGCTCCACCCT

**q-Real Time RT-PCR of human stemness marker genes**

1. **hOCT4**

F CAGTGCCCGAAACCCACAC

R GGAGACCCAGCAGCCTCAAA

1. **hSOX2-**

F GGG AAA TGG GAG GGG TGC AAA AGA GG

R TTG CGT GAG TGT GGA TGG GAT TGG TG

1. **hNANOG**

F TTTGGAAGCTGCTGGGGAAG

R GATGGGAGGAGGGGAGAGGA

1. **hLin-28**

F CAAAAGGAAAGAGCATGCAGAA

R ATGATCTAGACCTCCAGAGTTGTAGC

1. **hKLF4**

**F** GAAATTCGCCCGCTCCGATGA

**R** CTGTGTGTTTGCGGTAGTGCC

1. **hCXCR4**

F AGCATGACGGACAAGTACAGG

R GATGAAGTCGGGAATAGTCAGC

1. **hBCRP1**

F GGATGAGCCTACAACTGGCTT

R CTTCCTGAGGCCAATAAGGTG

1. **hNGFR**

F CCGAGGCACCACCGACAACC

R GGGCGTCTGGTTCACTGGCC

1. **hCD133 (PROM1)**

F CAG AGT ACA ACG CCA AAC CA

R AAA TCA CGA TGA GGG TCA GC

1. **hPou5f1*,***

F TTGGGCTAGAGAAGGATGTGGTT

R GGAAAAGGGACTGAGTAGAGTGTGG

1. **hp75NTR-**

F CGT ATT CCG ACG AGG CCA ACC

R CCA CAA GGC CCA CAA CCA CAG C

1. **hMusashi-1 (MSI1)**

F GAGGGTTCGGGTTTGTCACG

R GGCGACATCACCTCCTTTGG

1. **hNestin***,*

F AGAGGAAGAGCAGCAAGGCCATGAC

R TCCCTGACTCTGCTCCTTCTTCTTCAT

1. **PTEN**

F CCAGGACCAGAGGAAACCT

R GCTAGCCTCTGGATTTGA

1. **hGAPDH,**

F ATGGGGAAGGTGAAGGTCGG

R GACGGTGCCATGGAATTTGC
